# An update on treatments in myasthenia gravis

**DOI:** 10.1007/s00415-016-8359-x

**Published:** 2016-12-20

**Authors:** Lliwen A. Jones, Neil P. Robertson

**Affiliations:** Institute of Psychological Medicine and Clinical, Neurosciences Cardiff University, Cardiff, CF14 4XW UK

## Introduction

This month, we discuss three papers which publish the results of trials into potential treatment approaches to myasthenia gravis (MG). When treatment with cholinesterase inhibitors alone fails to control symptoms, treatment with steroids may be required, often at high doses which are often associated with adverse side effects both in the short and long term. All the studies explore treatments that may replace or reduce the need for steroids in MG and use the quantitative myasthenia gravis (QMG) score and prednisone dose requirement as primary or secondary outcomes. The first paper reports results of a randomised trial of routine thymectomy in non-thymomatous MG patients, the results of which may see this procedure incorporated into routine management of non-thymomatous MG. The second and third papers explore alternative steroid-sparing immunosuppressive agents. At present, the only medications shown to be effective in randomised placebo-controlled studies are azathioprine and cyclosporine, but both carry the risk of adverse reactions and have a requirement for close monitoring. The second study compares methotrexate and prednisone treatment to prednisone alone and the final study assesses effectiveness of leflunomide. All three studies represent a valuable addition to the literature on treatment of a condition that currently has limited alternatives to long-term immunosuppression with steroids, azathioprine, or cyclosporine.

## Randomized trial of thymectomy in myasthenia gravis

In this international multicentre randomised single-blind trial of thymectomy in patients with non-thymomatous MG, 126 participants were randomised to treatment with extended trans-sternal thymectomy plus alternate-day prednisone or treatment with alternate-day prednisone alone. The dual primary outcome was the time-weighted QMG score over 3 years and the time-weighted average required dose of prednisone over 3 years. Secondary outcomes addressed treatment safety and quality of life and focused on 36 treatment-associated complications and 29 symptoms associated with glucocorticoids.

Sixty-six participants were randomised to the thymectomy group of whom 58 underwent an extended trans-sternal thymectomy within 30 days of randomisation. The remaining eight participants declined surgery. Of the 60 participants randomised to the prednisone-only group, eight had a thymectomy outside the trial protocol. The trial was single-blinded as it was deemed unethical to perform a sham thymectomy. All participants wore a black, high-collared shirt to conceal any evidence of trans-sternal incisions to maintain rater blinding. Patients were treated with alternate-day prednisone starting at 10 mg, increasing in 10 mg steps to a maximum 100 mg on alternate days for participants not previously taking prednisone and 120 mg for those already taking prednisone, with the goal of achieving minimal-manifestation status. This was defined as having “no symptoms or functional limitations from myasthenia gravis, but there may be some weakness on examination of some muscles”. Once this was reached, the prednisone was reduced by 10 mg every 2 weeks until 40 mg was reached, with subsequent tapering by 5 mg a month to maintain the minimal-manifestation status. Pyridostigmine dose could not exceed 240 mg per day during the tapering phase and plasmaphersis or intravenous immunoglobulin was permitted in unstable patients.

In the thymectomy group, the time-weighted average QMG scores were significantly lower with an estimated difference in mean scores of 2.85 points. The average alternate-day prednisone dose was 44 mg in the thymectomy group compared to 60 mg in the prednisone-only group; also statistically significant. Minimal-manifestation status was reached in 67% of the thymectomy group compared to 37% of the prednisone-only group. There was no significant difference in treatment-associated complications between the groups.

*Comment* This study has demonstrated that routine thymectomy in patients with MG has a positive effect on QMG scores and reduces steroid dose required to maintain minimal-manifestation state. In a study assessing the validity of the QMG scale, patients with clinical improvement in symptoms scored 2.3 points lower; therefore, a score of 2.85 is likely to be associated with meaningful clinical improvement. This study provides class Ib evidence for performing thymectomy in MG and may result in this procedure being used with increasing frequency as a treatment option.

*Wolfe GI et al. (2016) N Engl J Med 375(6):511–21*

## A randomized controlled trial of methotrexate for patients with generalized myasthenia gravis

Methotrexate is a selective inhibitor of dihydrofolate and is commonly used as a steroid-sparing agent in a number of autoimmune diseases. This study is a multicentre randomised double-blind placebo-controlled trial designed to determine whether methotrexate has similar benefits in MG. Fifty participants were randomised to treatment with methotrexate and prednisone or treatment with matching placebo tablets and prednisone over 12 months. The primary outcome was the 9-month prednisone area under the dose-time curve (AUDTC), chosen as the authors felt that this most closely represents the experience of clinicians treating MG due to the need to vary prednisone dosing based on symptoms. The secondary outcomes focused on clinical status and included change in QMG score, MG Activities of Daily Living scale (MG-ADL), and MG Quality of Life scale (MG-QOL).

Twenty-five participants were randomised to each group. The methotrexate and placebo were over-encapsulated to ensure blinding of investigators, evaluators, and participants to treatment allocation. Participants were commenced on four tablets per week of either 2.5 mg of methotrexate or matching placebo, increasing by 2 tablets by every 2 weeks until a weekly dose of eight tablets was reached. A standardised protocol was then used to taper prednisone based on symptoms.

Forty-two participants completed the 12-month study. Eight participants withdrew from the study. One participant from the methotrexate group withdrew because of travel difficulties and seven participants from the placebo group as a result of adverse symptoms. All were included in the final analysis. There was no difference between groups of the primary or secondary outcomes. The study concluded that there was no steroid-sparing benefit for methotrexate in MG.

*Comment* The large confidence intervals reported and small sample sizes suggest that this study was underpowered. The sample sizes were calculated to detect the equivalent of a 31.4% reduction in the mean AUDTC over 9 months of treatment, so the study may have missed a moderate effect of methotrexate. There is a question about the appropriateness of the study duration, since a previous azathioprine study required more than 15 months before a reduction in prednisolone dose was detected. A further trial with a larger sample size and a longer duration of follow-up may be required before conclusively excluding methotrexate as a potential steroid-sparing immunosuppressant in MG.

*Pasnoor M et al. (2016) Neurology 87(1):57–64*

## Leflunomide treatment in corticosteroid-dependent myasthenia gravis: an open-label pilot study

Leflunomide is an immunosuppressant that blocks pyrimidine nucleotide biosynthesis and has been used in the treatment of rheumatoid arthritis. Its active metabolite, teriflunomide has also been investigated as a potential treatment for multiple sclerosis and found to be associated with lower relapse rate and less disability accrual compared to placebo. This third paper reports the findings of an open-label pilot study of leflunomide treatment in MG. The primary purpose of the study was to examine the efficacy of leflunomide in MG using the QMG and MG-ADL scores. The study was not designed to assess the steroid-sparing effects of the drug, although a comparison was made of prednisone doses at the beginning and end of the study.

Fifteen participants were enrolled in the study. All had undergone thymectomy and were dependent on steroid treatment. They were assigned to one of two groups based on the duration of previous steroid treatment. The first group consisted of 11 participants treated for longer than 6 months, and the second group had 4 participants treated for less than 6 months. Both groups were given prednisone and leflunomide which were administered orally at 20 mg daily for 6 months. There was no protocol for prednisone dosing which was done at the discretion of the treating clinician.

Following 6 months of treatment, there was a significant improvement in QMG score by 3 points or more in 11 participants and an improvement in ADL score in 10 participants. Twelve participants had their prednisone dose reduced with a reduction in average dose from 24.3 to 12.3 mg per day which statistically significant. No major adverse reactions were reported. Three participants reported minor side effects, including diarrhoea, transient thrombocytopenia, gastric disturbance, hair thinning, and rash.

*Comment* This is a small but promising pilot study demonstrating that leflunomide may be an efficacious and safe steroid-sparing immunosuppressant for MG. As was the case in the second study, a longer duration of follow-up would have been desirable given the previous 15-month treatment duration required to establish the benefits of azathioprine. A larger, placebo-controlled study over a longer time period is planned by the authors to confirm the findings of their pilot study and to further assess the safety profile of leflunomide when used in patients with MG.

*Chen P* *et al*. *(2016) J Neurol 263(1):83*–*8*

